# Preparation of Cu^2+^/TA/HAP composite coating with anti-bacterial and osteogenic potential on 3D-printed porous Ti alloy scaffolds for orthopedic applications

**DOI:** 10.1515/biol-2022-0826

**Published:** 2024-02-08

**Authors:** Xu Haitao, Li Siyuan, Guo Sutong, Guo Yu, Xu Peirong, Wang Ling, Ding Yujian, Feng Dehong

**Affiliations:** Wuxi People’s Hospital of Nanjing Medical University, Wuxi 214000, Jiangsu, China; School of Chemical and Material Engineering, Jiangnan University, Wuxi 214000, Jiangsu, China; Wuxi No. 5 People’s Hospital, Wuxi 214000, Jiangsu, China

**Keywords:** 3D printed porous titanium scaffolds, composite coating, artificial bone grafts, hydroxyapatite, chitosan, nanoparticles, osteogenesis

## Abstract

Because of stress shielding effects, traditional titanium (Ti) alloy scaffolds have a high elastic modulus, which might promote looseness and bone disintegration surrounding the implant, increasing the likelihood of a second surgery. In contrast, 3D-printed porous Ti alloy scaffolds can reduce the scaffold weight while enhancing biocompatibility. Further, these scaffolds’ porous nature allows bone tissue ingrowth as well as strong pore connectivity, which can improve nutrient absorption. Nevertheless, bare Ti alloy implants may fail because of inadequate bone integration; hence, adding a coating on the implant surface is an effective technique for improving implant stability. In this study, a composite coating comprising hydroxyapatite (HAP), chitosan (CS), tannic acid (TA) and copper ions (Cu^2+^) (Cu^2+^/TA/HAP composite coating) was prepared on the surface of 3D printed porous Ti alloy scaffolds using electrophoretic deposition. Using the standard plate count method, Live/Dead bacteria staining assay, FITC Phalloidin and 4′,6-diamidino-2-phenylindole staining assay, and live/dead staining of cells we determined that the composite coating has better antibacterial properties and cytocompatibility as well as lower cytotoxicity. The Alkaline Phosphatase assay revealed that the coating results showed good osteogenesis potential. Overall, the composite coatings produced in this investigation give new potential for the application of Ti alloys in clinics.

## 1 Introduction

Because of the aging population, the number of bone grafting procedures required by bone abnormalities has increased globally in recent years [1]. Trauma, tumor excision, or congenital disease can all cause bone abnormalities. The current strategies for treating bone defects include autologous and allogeneic bone grafts. Nevertheless, these techniques are severely restricted by donor site morbidity, a scarcity of autologous bone, and a mismatch in defect shape. As a result, artificial bone substitutes have received a lot of attention for treating bone abnormalities [2,3]. Because Ti alloy is biocompatible and has no harmful or adverse effects on the human body, it has been frequently employed in the clinic [4]. Ti alloy also has excellent corrosion resistance and high mechanical strength [5]. However, traditional Ti alloy scaffolds have a greater elastic modulus, which might result in looseness and bone disintegration surrounding the implant due to stress shielding effects. As a result, the implant components may sink and loosen, raising the likelihood of a second operation [6]. Compared with the traditional Ti alloy scaffolds, three-dimensional (3D) printed porous Ti scaffolds can reduce the scaffold weight while enhancing their biocompatibility. Further, porous structure with appropriate-sized pores permits bone tissue ingrowth, and strong pore connection enhances nutrient absorption [7].

Nevertheless, implants made of bare Ti alloy might fail owing to poor incorporation into the surrounding bone. Because the contact surface between the host bone and the implant is unstable, issues like aseptic loosening [8], mechanical damage, and even implant detachment [9] can occur. To remove the implant, a second surgery is required, which can result in further injury to the patient. To eliminate this challenge, it is necessary to improve implant stability. Applying a coating on the implant surface is an effective method to achieve this. There have been various aspects of implant coatings that have been studied in the past, including coating material and coating preparation methods. However, past studies have paid more attention to coatings prepared by a single material. There is a lack of research on composite coatings prepared by various materials.

Since it can imitate the nanoscale architecture and chemical makeup of the natural bone mineral, hydroxyapatite (HAP) coating is often used to increase the osteoconductivity and osteointegration of Ti alloy scaffolds [10,11,12,13]. Li et al. [14] applied a polydopamine (PDA)-assisted hydroxyapatite coating (HAP/PDA) onto 3D-printed porous titanium alloy scaffolds, which enhanced osteointegration and significantly promoted bone regeneration. Further, the problem of implant infection caused by bacteria can also lead to the failure of Ti alloy implants, resulting in serious complications and even death [15,16]. As a result, it is critical that implants be endowed with antimicrobial qualities. Stevanović et al. [17] utilized an electrophoretic deposition (EPD) technique to successfully prepare a composite coating comprising gentamicin (Gent), chitosan (CS), and HAP; this composite coating showed good antibacterial activity and low cytotoxicity. In the follow-up study, Stevanović et al. [18] further evaluated the effect of the coating (HAP/CS/Gent) on Ti, and found that the composite coating demonstrated good antibacterial properties and biocompatibility. Inspired by these findings, we aimed to construct a composite coating on 3D printed porous Ti alloy scaffolds to improve their antibacterial properties and osteogenesis potential.

In this study, a composite coating comprising HAP, CS, tannic acid (TA) and copper ions (Cu^2+^) (Cu^2+^/TA/HAP composite coatings) was prepared on the 3D-printed porous Ti alloy scaffolds surface. Because it is biocompatible, biodegradable, nontoxic, and has strong antibacterial action, CS is an appealing natural polymer in biomedicine [19]. Chitosan, being a biopolymer, has a great capacity of film formation [20], implying that the presence of CS can boost composite coating adhesion [21]; additionally, CS has been found to be effective in the tissue repair process [22]. The chitosan-containing templates were previously introduced as suitable materials with both antibacterial and bone regeneration properties [23]. TA surface modification is functionally identical to the commonly used PDA coating. Additionally, it overcomes PDA’s drawbacks such as interference in ligand measurement, high cost, and alkaline pH [24]. TA is a polyphenolic biomolecule with good antibacterial properties; it can complex with a variety of metal ions to form stable complexes [25]. The addition of Cu^2+^ can further improve the antibacterial characteristics of the coating [26,27]. EPD is an appropriate technology for producing composite coatings and may be used to produce coatings on the surfaces of complicate shaped objects [28,29]. Since CS is positively charged under acidic circumstances [30], it may be utilized to produce Cu^2+^/TA/HAP composite coatings (CS@NP) on porous Ti alloy scaffolds that have been 3D printed. In fact, the gelation process, porosity and finding the final form of chitosan with suitable features for tissue regeneration strongly depends on the pH of the environment and the gelation time [31]. After preparing the coating, a series of characterization methods were used to evaluate the tissue repair, antimicrobial, and biocompatibility properties of the coating.

## 2 Materials and methods

### 2.1 Materials

The 3D printed porous Ti alloy scaffolds were provided by Wuxi People’s Hospital. The Ti sheet used was 2 cm in length, 1 cm in width, and 0.1 mm in thickness. The samples were polished with 2,000 grit emery sheets and ultrasonically cleaned with 100% ethanol and acetone for 15 min, followed by air drying. Ethanol (AR), acetic acid (AR), and acetone (AR) were obtained from Sinopharm Chemical Reagent Co., Ltd (Shanghai, China). HAP (nanopowder) was obtained from Sigma-Aldrich. TA (95%) and chitosan powder (low viscosity) were purchased from Aladdin Reagent Co., Ltd (Shanghai, China). Copper (II) chloride dihydrate (99%) was obtained from J&K Scientific. Fetal bovine serum (FBS, Hyclone) and penicillin-streptomycin were obtained from Bomeida Reagent Instrument Co., Ltd (Suzhou, China). Fluorescein diacetate (FDA) and propidium iodide (PI) were purchased from Trivd Biotechnology Co., Ltd (Wuxi, China).

## 3 Methods

### 3.1 Synthesis and characterization of nanoparticles (NPs)

TA/HAP NPs were prepared by dispersing HAP (2 g) into 50 mL of TA aqueous solution (5 mg/mL), and were then separated by centrifugation at 9,000 rpm for 10 min. The supernatant was eliminated and the solid was dried to obtain the TA/HAP NPs. The TA/HAP NPs were dispersed into 50 mL of aqueous copper(II) chloride dihydrate solution (5 mg/mL), and separated by centrifugation at 9,000 rpm for 10 min; the liquid was drained out and the solid was dried to obtain Cu^2+^/TA/HAP NPs. Transmission electron microscopy (TEM) (Hitachi JEOL JEM-2100, Japan) and thermogravimetric analyses (TGA) were utilized to characterize the obtained NPs.

#### 3.1.1 Preparation and characterization of composite coatings

A coating was first prepared on the Ti alloy sheet using EPD to simplify the experiment. First, the acetic acid aqueous solution (volume ratio, 1%) was prepared. This was mixed with 1% CS by mass to obtain CS acetic acid aqueous solution. In previous studies, the EPD of TA/HAP/CS coatings has been carried out using ethanol, isopropanol, methanol, as well as mixed ethanol/water baths [32,33,34]. In this study, to suppress the electrolysis of water, we selected a mixed ethanol/chitosan acetic acid aqueous solution bath (volume ratio 9:1). To obtain a uniform coating, a cylindrical ring of stainless steel was used as the anode, Ti alloy sheet was used as the cathode, and deposition was performed for 5 min at 20 volts DC. The EPD solution without NPs was used as the control group (the obtained sample is abbreviated as Ti-CS, Ti alloy sheet with CS coating). HAP NPs were added to the above mixed solution, and ultrasonic dispersion was performed to prepare the HAP EPD solution (1 mg/mL) (Ti-CS@HAP, Ti alloy sheet with CS@HAP coating); the Cu^2+^/TA/HAP EPD solution (1 mg/mL) (Ti-CS@NP, Ti alloy sheet with CS@NP coating) was prepared as described above. The method of coating on the 3D printed porous Ti alloy scaffolds is also the same as described above. Fourier transform infrared spectroscopy (FT-IR) (Nicolet iS50, USA), X-ray diffraction (XRD) (D8, Germany), scanning electron microscopy (SEM) (Hitachi S-4800, Japan), and energy dispersive spectroscopy (EDS) were utilized to characterize the prepared coatings. An elemental analyzer (Vario EL, Elementar, Germany) was utilized for elemental analysis.

#### 3.1.2 Bacterial culture

For bacterial culture, 100 μL of *Escherichia coli* (*E. coli*) and *Staphylococcus aureus* (*S. aureus*) were respectively inoculated in 20 mL of LB medium, and the flasks were shaken for 12 h in an incubator at a constant temperature of 37°C (THZ-98C, Shanghai) and 120 rpm. (The LB broth was added with tryptophan, sodium chloride, yeast extract, and deionized water at a ratio of 2:2:1:200.) Bacterial concentration was determined by a microplate reader (MD SpectraMax 190, USA) at 540 nm wavelength, and adjusted to 10^8^ cells/mL.

#### 3.1.3 Antimicrobial properties of the coatings

A standard plate count technique was utilized to examine the coatings’ antibacterial characteristics. After UV sterilization for 30 min, the samples were submerged vertically in the prepared bacterial solution and cultured in an incubator shaker at a constant temperature. After incubation, non-adherent bacteria were removed by washing thrice with 0.9 wt% NaCl solution. Every sample was then sonicated for 30 min in 10 mL of 0.9 wt% NaCl solution to liberate the adherent bacterial cells. Following that, 100 μL of sonicated bacterial solution was taken and diluted 10, 100, and 1,000 times, respectively. Finally, 100 μL of the diluted bacterial solution was distributed on an agar plate and incubated for 24 h at 37°C (The ratio of nutrient agar and deionized water in the agar solution was 8:25). Afterwards, pictures were taken of the bacterial colonies that had grown on the agar plates.

The bacteria dispersion on the composite coating was determined using the Live/Dead staining technique. The samples were put in 96-well plates, and each well was filled with 200 L of bacterial suspension (108 CFU/mL, PBS). Following 10 h of incubation at 37°C, the samples were gently rinsed and treated with a mixture of SYTO 9 (6 µmol/L) and PI (30 µmol/L) (L7012 kit) for 10 min. After the treatment, an upright fluorescence microscope (Nikon 80i, Japan) was utilized to observe the coatings and capture the images.

#### 3.1.4 Cell culture

The cytocompatibility and osteogenesis-supporting potential of the implants were determined employing mouse preosteoblast cells (MC3T3-E1). In sterile tissue culture flasks, the cells were grown at 37°C in complete medium (made using RPMI-1640 medium, fetal bovine serum, and penicillin-streptomycin in a ratio of 90:10:1) in a 5% CO_2_ humidified environment.

#### 3.1.5 Cell adhesion and morphology

MC3T3-E1 cells were planted at a density of 5 × 10^4^ cells/well in 24-well culture plates on the samples (bare Ti alloy, Ti-CS, and Ti-CS@NP) and cultivated for 3 days. After that, the cells were fixed by treatment with glutaraldehyde for 30 min, followed by successive dehydration with 70, 80, 95%, and absolute ethanol. Finally, SEM was utilized to observe the cell morphology on the coating surface. The above-mentioned method was used to characterize cell adhesion characteristics on scaffold coatings.

FITC phalloidin (obtained from Solarbio) and 4′,6-diamidino-2-phenylindole (DAPI) were utilized to stain the F-actin and nucleus of the cells, respectively, to visualize the cytoskeleton of cells on distinct samples. In brief, the cells were planted on the samples at a density of 5 × 10^4^ cells/well in 24-well culture plates and cultivated at 37°C for 24 h in a 5% CO_2_ humidified environment. After removing the medium and rinsing thrice with PBS, paraformaldehyde at a concentration of 4% was used to fix the cells in PBS for 15 min, washed thrice with PBS, permeabilized using 0.5% Triton-X-100 for 5 min at ambient temperature, and finally rinsed with PBS at ambient temperature to wash off the excess Triton-X-100. FITC Phalloidin was used to stain F-actin for 30 min at ambient temperature in the dark, and cell nuclei were stained with DAPI for 30 s as per the manufacturer’s instruction. Finally, an upright fluorescence microscope (Nikon 80i, Japan) was used to observe and record the cytoskeleton.

#### 3.1.6 Cytotoxicity assay

Copper ions exert a bactericidal effect, but can also cause damage to cells [35]. Therefore, characterizing the cytotoxicity of the coating synthesized above is necessary. FDA and PI were utilized to perform Live/Dead staining. In brief, cells were planted at a density of 5 × 10^4^ cells/well in 24-well culture plates on the samples (bare Ti alloy, Ti-CS, and Ti-CS@NP) and cultivated for 48 h. Afterwards, FDA (5 mg/mL in acetone) was used to stain live cells, while PI (1 mg/mL in PBS) was used to stain dead cells. Each treatment was assessed in triplicate. Finally, an upright fluorescence microscope (Nikon 80i, Japan) was utilized to observe and record the adhesion morphology of the cells on the substrate surface and the proportion of live/dead cells. Live cell number and total coverage area were used to characterize the cytotoxicity of the coatings quantitatively.

#### 3.1.7 Osteogenic differentiation

An Alkaline Phosphatase Assay Kit (ALP, purchased from Beyotime, China) was used to confirm the osteogenesis of MC3T3-E1 cells on the samples. Cells were seeded on sterilized samples (bare Ti alloy, Ti-CS, and Ti-CS@NP) at a density of 5 × 10^4^cells/well in 24-well culture plates and cultivated in a 5% CO_2_ humidified environment at 37°C for 7 days. After removing the medium, 4% paraformaldehyde (PFA) solution was used to fix the cells on the samples for 20 min, followed by washing with PBS. An appropriate amount of BCIP/NBT alkaline phosphatase color reagent was added (covering the sample), incubated in the dark for 1 h, and rinsed with PBS. Finally, imaging was performed using a light microscope (Nikon, Japan).

## 4 Results and discussion

### 4.1 Synthesis and characterization of NPs

**Figure 1:**
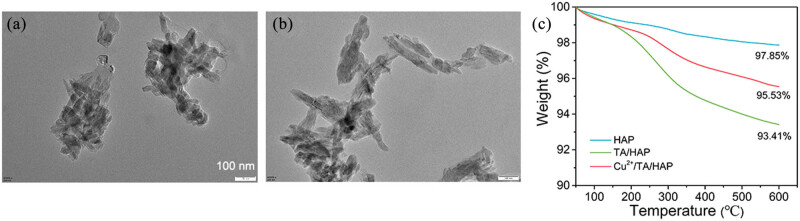
(a) TEM image of HAP NPs. (b) TEM image of TA/HAP NPs. (C) TGA curves of HAP, TA/HAP, and Cu^2+^/TA/HAP NPs.

TEM and TGA were used to characterize the NPs. As shown in Figure 1a, HAP NPs exhibit a typical rod-like structure. TA/HAP NPs also exhibit a rod-like structure as shown in Figure 1b. Thus, addition of TA does not affect the morphology of the NPs. Figure 1c illustrates the TGA curves of HAP, TA/HAP, and Cu^2+^/TA/HAP NPs. The mass loss of HAP, TA/HAP, and Cu^2+^/TA/HAP NPs was 2.15, 6.59, and 4.47%, respectively. The mass loss of all NPs was lower by 10%, indicating that the thermal stability of the NPs is better, facilitating their application in the clinic.

Free TA and HAP show electrostatic interaction; further, TA’s phenolic hydroxyl group has the ability to chelate HAP’s calcium ion. After adding copper ions, the remaining phenolic hydroxyl group of TA can be chelated. Copper ions, and the hydroxyl groups in HAP also exert a coordination effect. This double coordination effect fixes copper ions on the surface of composite particles, imparting functional particles with antibacterial properties, while HAP plays a role in bone repair function.

### 4.2 Preparation and characterization of composite coatings

According to available literature [17], CS can become soluble in water at pH values below 6.7 [36].

(1) \[\text{CS}\mbox{--}{\text{NH}}_{\text{2}}{\text{+ H}}_{\text{3}}{\text{O}}^{\text{+}}\leftrightarrow \text{CS}\mbox{--}{{\text{NH}}_{\text{3}}}^{\text{+}}{\text{+ H}}_{\text{2}}\text{O}.]\]

During the EPD, the reactions occurring at the cathode and anode are as follows [37].

(2) \[\text{cathode:}\hspace{.25em}{\text{2H}}_{\text{2}}O+{\text{2e}}^{-}\to {\text{H}}_{\text{2}}+\text{2OH},]\]

(3) \[\text{anode:}\hspace{.25em}{\text{2H}}_{\text{2}}O+4{\text{H}}^{+}\to {\text{O}}_{\text{2}}+4\text{e}.]\]

Since the HAP surface was protonated, it was positively charged in the acidic solution; as a result, HAP particles were pushed to the cathode (Ti alloy sheet and scaffold) by the electric field, where they deprotonated and then the coating was deposited [35].

(4) \[\text{CS}\mbox{--}{{\text{NH}}_{\text{3}}}^{+}+{\text{OH}}^{-}\leftrightarrow \text{CS}\mbox{--}{\text{NH}}_{\text{2}}+{\text{H}}_{\text{2}}\text{O}.]\]

**Figure 2:**
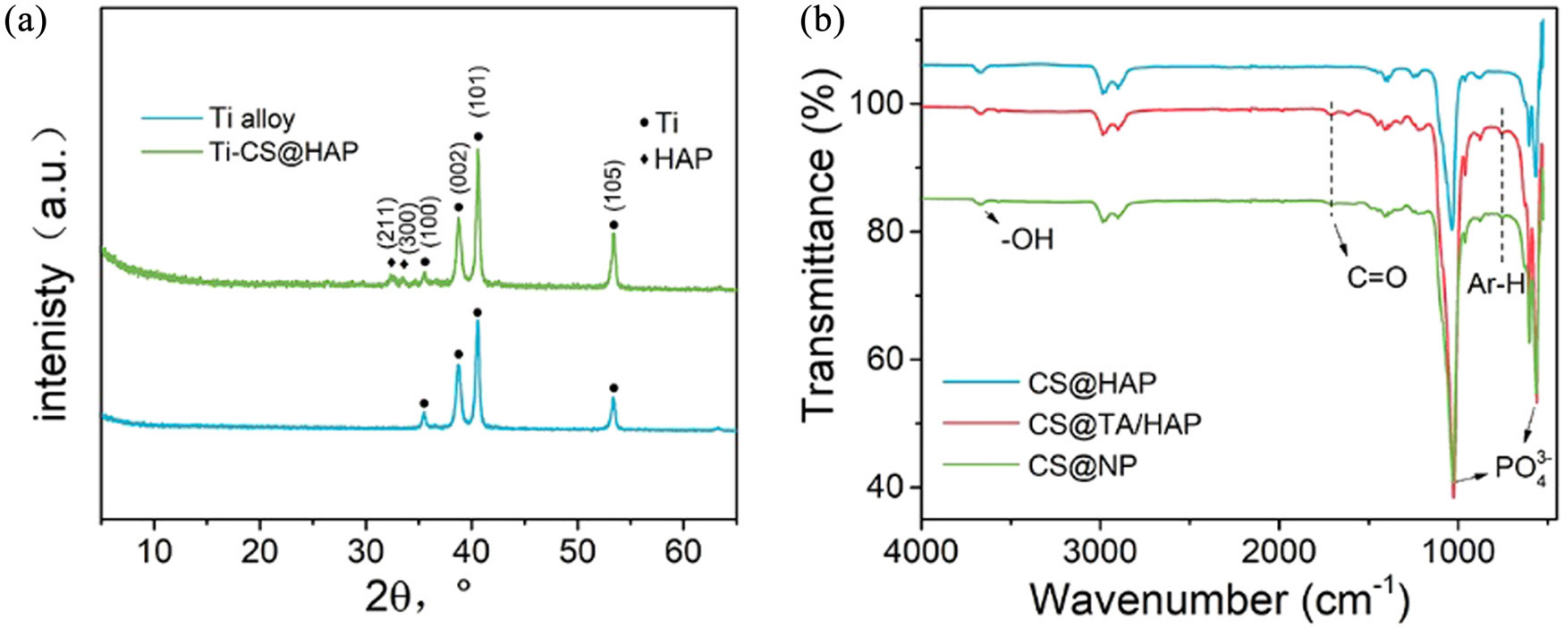
(a) XRD patterns of Ti alloy and coating of CS@HAP on a Ti sheet. (b) FT-IR spectra of CS@HAP, CS@TA/HAP, and CS@NP.

After drying, a surface layer was prepared on the Ti alloy sheet surface. FT-IR and XRD were used to determine the makeup of the surface layer on the coated Ti alloy sheet. Figure 2a illustrates XRD patterns for bare Ti alloy sheets and coating the CS@HAP on the Ti alloy sheet respectively. The characteristic peaks of bare Ti alloy appear at 35.5°, 38.7°, 40.6°, and 53.5°. The characteristic diffraction peaks of HAP appear at 32.2° and 33.5°, corresponding to the HAP crystal planes (211) and (300), showing that the coating was successfully loaded with HAP.

The FT-IR spectra of CS@HAP, CS@TA/HAP, and CS@NP on the Ti alloy sheet are shown in Figure 2b. A wide band was found in the range from 3,000 to 3,400 cm^−1^, corresponding to the stretching vibration of the –OH group originated from HAP in the surface layer of CS@HAP, CS@TA/HAP, and CS@NP. Further, the bands detected at 562 and 601 cm^−1^ were corresponding to the phosphate group’s asymmetric and symmetric deformation modes, and the band detected at 1,015 cm^−1^ was ascribed to the phosphate group’s v3 vibration mode [38,39] originating from HAP. These findings indicate that the coating was successfully loaded with HAP. The band at 1,720 cm^−1^ that corresponds to the stretching vibration of the −C═O group originated from TA in the surface layer of CS@TA/HAP and CS@NP. Further, the peak at 755 cm^−1^, corresponded to the bending vibration of Ar-H in TA. These evidence indicate that the coating was successfully loaded with TA.

**Figure 3:**
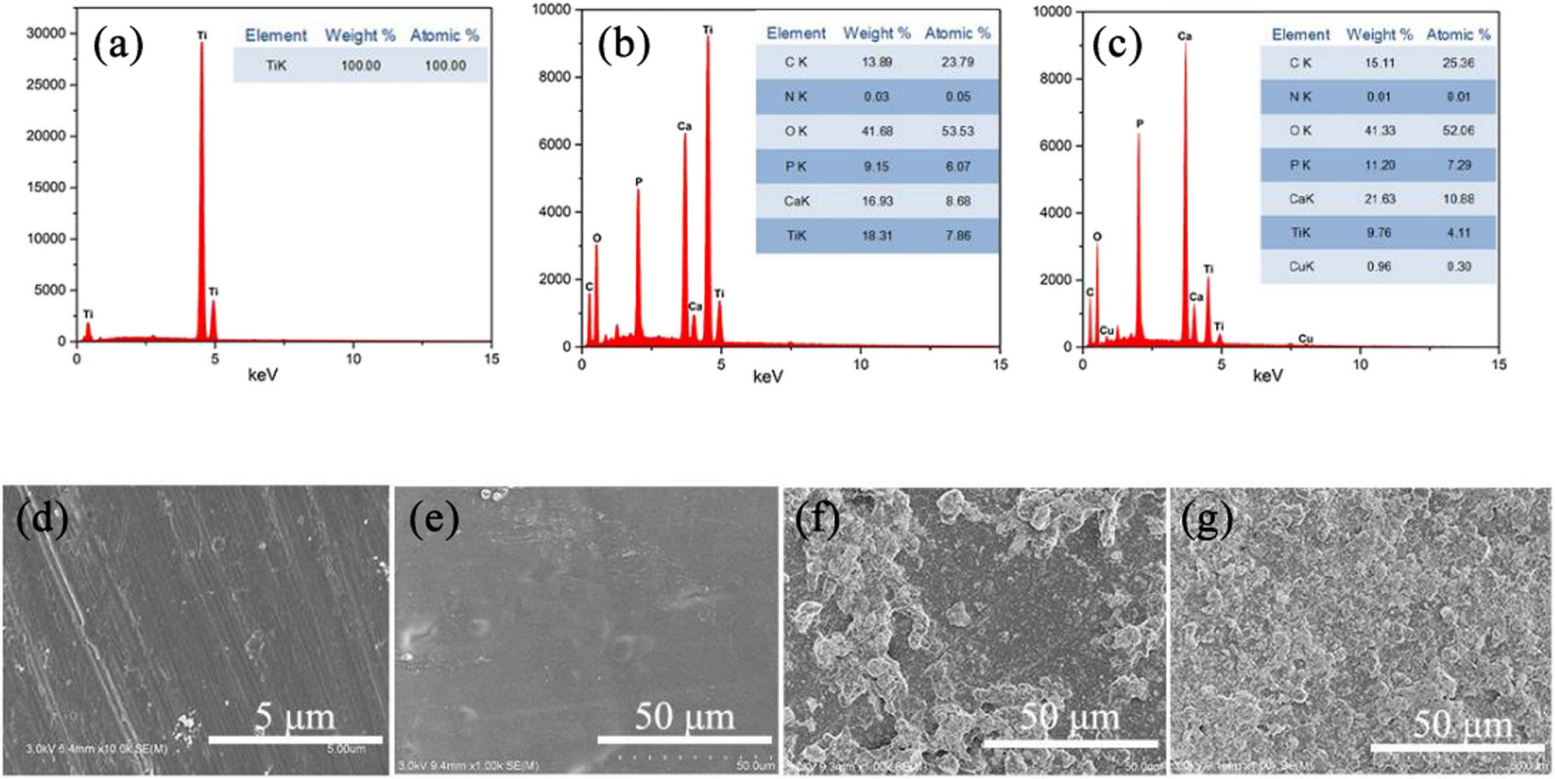
EDS data for (a) bare Ti sheet, (b) composite coating of CS@HAP on Ti sheet, and (c) composite coating of CS@NP on Ti sheet. SEM images of (d) bare Ti sheet, (e) coating of CS on Ti sheet, (f) composite coating of CS@TA/HAP on Ti sheet, and (g) composite coating of CS@NP on Ti sheet.

As demonstrated in Figure 3a–c, the elemental makeup of the coatings on the Ti alloy sheet surface was further analyzed using EDS. In Figure 3b, the peaks of C and O appeared, indicating that CS was prepared on the Ti alloy sheet surface with success; the peaks of Ca and P demonstrate the successful deposition of HAP on the Ti alloy surface, providing the same conclusion as the above characterization. The appearance of the Cu peak in Figure 3c indicates that the CS@NP was successfully prepared on the Ti sheet.

**Figure 4:**
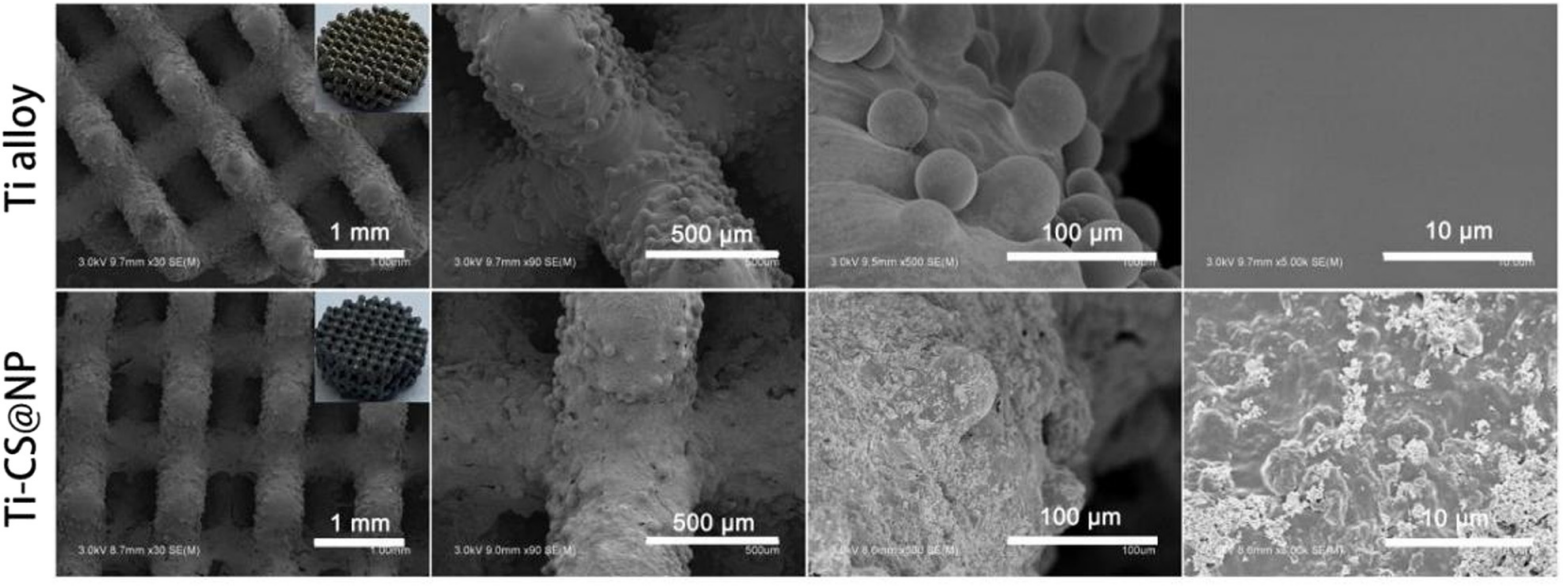
SEM images of bare 3D printed porous Ti alloy scaffolds and scaffolds with the CS@NP coating at different magnifications. Inset: digital image of the bare 3D printed porous Ti alloy scaffolds and scaffolds with the CS@NP coating.

SEM was applied to characterize the composite coatings’ morphology on the Ti sheet surface and presented in Figure 3d–g. Clear grinding marks can be seen in Figure 3d. After CS deposition on the Ti alloy sheet surface, the grinding marks disappeared and a flat and smooth coating was obtained. As presented in Figure 3f, the accumulation of large particles was observed, forming a relatively rough surface. Compared with the composite coating of CS@TA/HAP, the composite coating of CS@NP was relatively smoother. This may be attributed to the reduced potential of the HAP by the surface addition of Cu^2+^/TA (as shown in Table 1), reduced interaction between HAP particles, and more uniform dispersion of HAP. The morphology of 3D printed porous Ti alloy scaffolds without and with the CS@NP coating is shown in Figure 4. The surface morphology of the stent resembles that of the Ti alloy sheet before and after the coating preparation.

**Table 1:** Zeta potentials of CS, CS@HAP, and CS@NP mixtures (10% H_2_O, 90% EtOH)

Component	CS	CS@HAP	CS@NP
Zeta Potential (mV)	20.4 ± 1.2	24.6 ± 3.9	21.4 ± 7.5

### 4.3 Antimicrobial properties of the coatings

**Figure 5:**
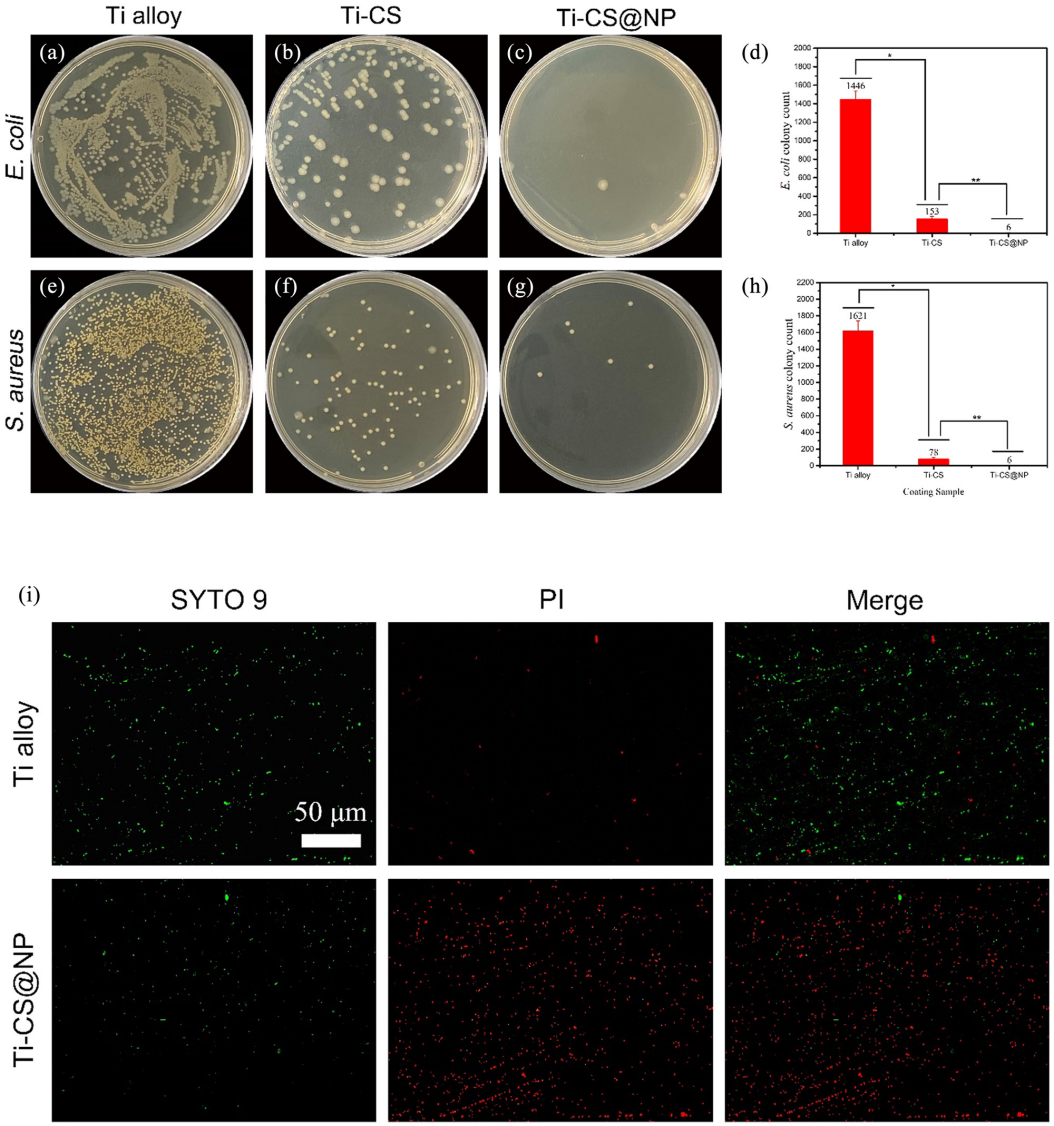
Pictures of *E. coli* and *S. aureus* colonies incubated on agar plates with bare Ti alloy (a, e), Ti alloy with CS coating (b, f), and Ti alloy with CS@NP coating (c, g). Quantitative evaluation of *E. coli* (d) and *S. aureus* (h) colonies (*p < 0.05, **p < 0.01, calculated by three parallel groups). Fluorescence images (i) showing the live/dead bacteria on bare Ti alloy and Ti-CS@NP.

Standard plate count method and live/dead staining were used to investigate the coating’s antibacterial activity. Figure 5a–h illustrates the antibacterial performance of the coating using the plate colony counting method. The quantity of *E. coli* colonies on the agar plate of the bare Ti alloy (Figure 5a) was very large, indicating that the bare Ti alloy has little antibacterial ability. Compared to the bare Ti alloy, the quantity of colonies on the agar plate of Ti-CS (Figure 5b) was obviously decreased, indicating that the CS coating has certain antibacterial properties. The quantity of colonies on the agar plate of Ti-CS@NP (Figure 5c) was reduced further, proving that the CS@NP coating has good antibacterial properties against *E. coli.* Quantitative evaluation of the *E. coli* colonies (Figure 5d) clearly showed that Ti-CS@NP group had the fewest *E. coli* colonies, and that CS@NP shows good antibacterial properties against *E. coli*. Based on Figure 5(e–h), we can obtain a similar conclusion as above that the CS@NP coating has strong antibacterial activities against *S. aureus.* These findings indicate that the CS@NP coating has good antibacterial properties overall.

To further investigate the antibacterial characteristics of the coating, a live/dead bacteria staining technique was employed. Live bacteria showed green fluorescence upon staining with SYTO 9 whereas dead bacteria were stained red by PI (Figure 5i). A great quantity of live bacteria was found on the bare Ti alloy with very few dead bacteria. Compared with that on bare Ti alloy, the quantity of live bacteria on the Ti-CS@NP was obviously decreased and the number of dead bacteria was increased significantly. These observations support the conclusion that the CS@NP coating has good antibacterial properties.

Cu^2+^ has an excellent antibacterial effect, as it can combine with the sulfhydryl group and alter bacterial cell membrane permeability as well as produce reactive oxygen species, which oxidize bacterial proteins and degrade DNA, resulting in bacterial death; further, Cu^2+^ can interfere with cell division and replication, thus affecting bacterial DNA synthesis. Cu^2+^ can also change the structure and function of bacterial cell wall proteins and cause cell rupture [40,41,42].

### 4.4 Cell adhesion and morphology

**Figure 6:**
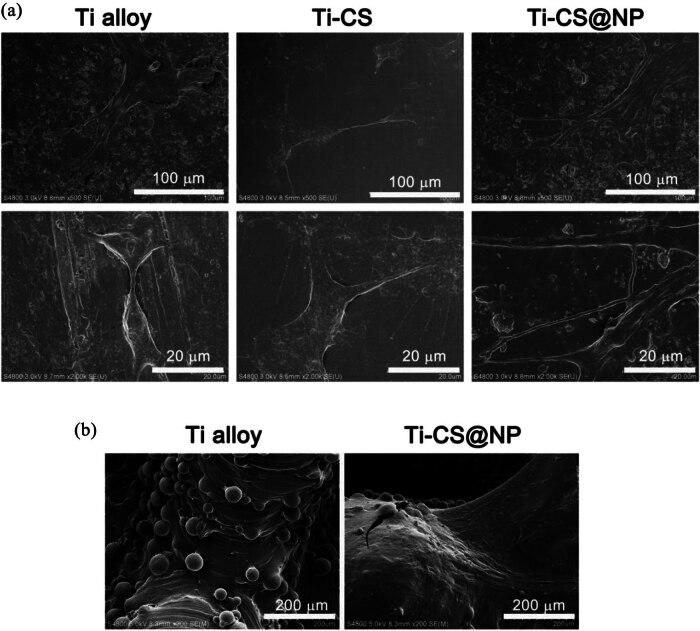
SEM images of cells on the Ti alloy sheet samples surface (bare Ti alloy, Ti-CS, and Ti-CS@NP) (a). SEM images of cells on the 3D-printed porous Ti alloy scaffolds surface (bare Ti alloy and Ti-CS@NP) (b).

MC3T3-E1 cells could be seen growing on the Ti alloy sheet sample surfaces (bare Ti alloy, Ti-CS, and Ti-CS@NP) using SEM (Figure 6a). In detail, cells curl up and spread poorly on the surface of bare Ti alloy, indicating its poor cytocompatibility. Cells on the Ti-CS surface displayed lamellipodia extensions, which were better than the cells spread on the bare Ti surface. On the Ti-CS@NP surface, the cells displayed more lamellipodia extensions and more cells adhered to the surface suggesting that the coating promotes cell adhesion and migration. This indicates that the CS@NP coating has good cytocompatibility. The 3D-printed porous Ti alloy scaffolds also showed similar results (Figure 6b), with more cell adhesion on the Ti-CS@NP surface compared to that on the bare Ti alloy.

**Figure 7:**
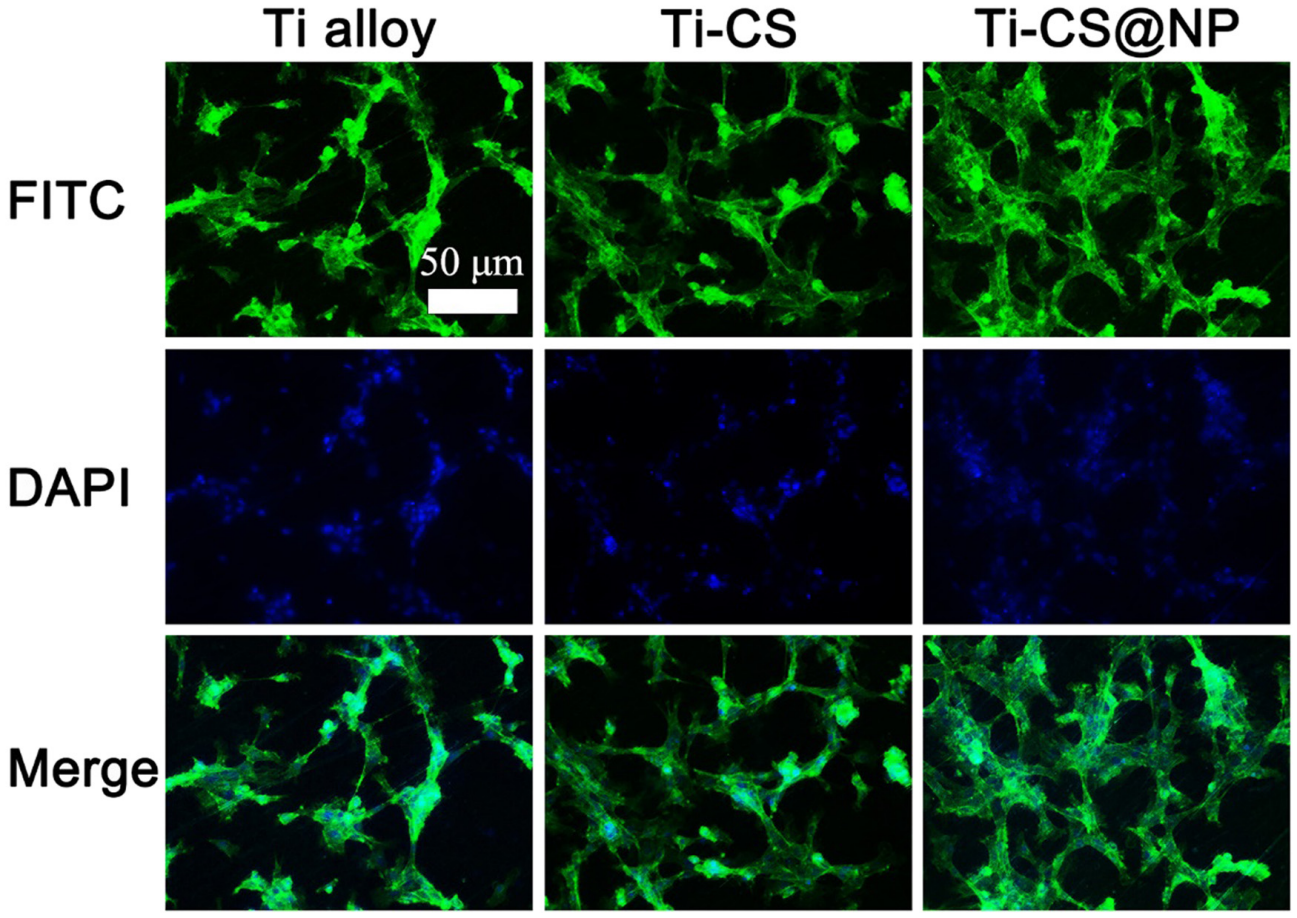
Fluorescence images of cells stained with FITC-Phalloidin and DAPI. Green fluorescence indicates F-actin and blue fluorescence indicates cell nuclei.

Cells were stained with FITC Phalloidin and DAPI to visualize F-actin (green fluorescence) and cell nuclei (blue fluorescence) (Figure 7). As observed from the merged image, more cells were found on the Ti-CS@NP surface and the cytoskeleton of cells on the Ti-CS@NP surface was also spread more, providing further evidence that this coating facilitates cell attachment and spreading.

Implants’ surface properties affect the conformation and orientation of cell adhesion proteins. These can cause changes in the complicated cell adhesion process, which includes cell attachment, cell dissemination, and cytoskeletal architecture, activating the subsequent cell response cascade [43]. As a result, favorable cell adhesion and spread promote cell survival, proliferation, differentiation, and osseointegration.

### 4.5 Cytotoxicity assay

**Figure 8:**
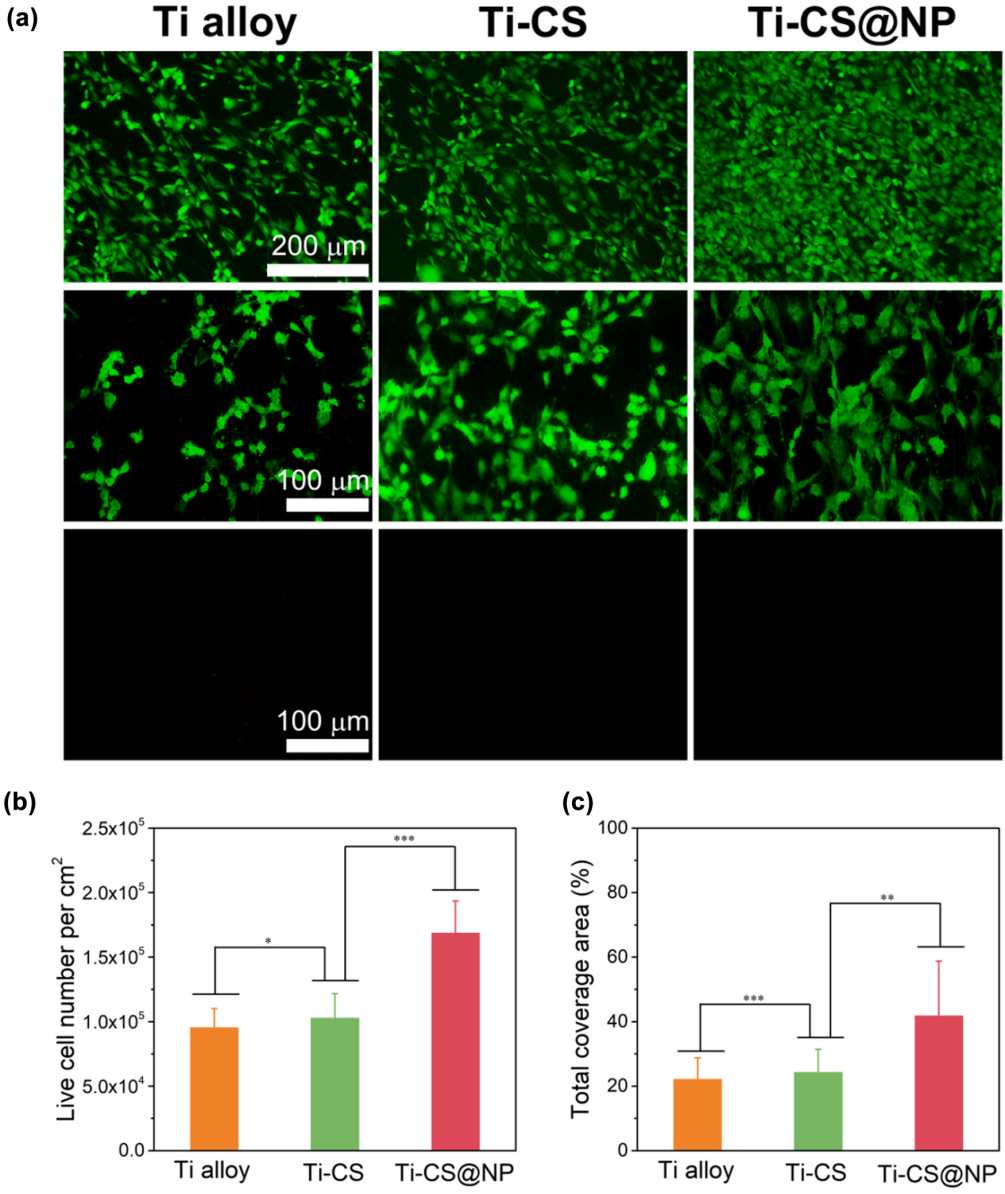
Live/dead staining of cells on the Ti alloy sheet samples surface (bare Ti alloy, Ti-CS, and Ti-CS@NP) (a). Green fluorescence represents viable cells and red fluorescence represents dead cells. The quantity of live cells on the Ti alloy sheet samples surface (b). The total coverage area of cells on the Ti alloy sheet samples surface (c). (*p < 0.05, **p < 0.01, ***p < 0.001, three pictures of different positions were selected).

Live cells tagged with FDA exhibit green fluorescence, whereas dead cells tagged with PI present red fluorescence as shown in Figure 8a. The images show that the cell morphology on the Ti-CS@NP surface spread better and displayed more lamellipodia extensions than that on bare Ti alloy and Ti-CS surfaces, indicating that the CS@NP coating had better cytocompatibility. Based on the red fluorescence images, there were only a few scattered dead cells on the bare Ti alloy surface, whereas on the Ti-CS and Ti-CS@NP surfaces, almost no dead cells could be observed. These findings indicated that CS and CS@NP coatings cause little harm to cells. Therefore, we concluded that the CS@NP coating prepared above has little cytotoxicity and good cytocompatibility. The quantitative data presented in Figure 8b and c illustrate this more clearly. The live cell number on the bare Ti alloy surface, Ti-CS and Ti-CS@NP increased sequentially, and the quantity of cells on the Ti-CS@NP surface was almost 1.9 times that on the bare Ti alloy, similar to the fluorescence images, proving that the CS@NP coating has little cytotoxicity. Further, the overall cell coverage area on the Ti-CS@NP surface is twice that on the bare Ti alloy, which indicated that this coating has better cytocompatibility.

To overcome the growing problem of bacterial resistance to antibiotics [44], metal ions such as copper, silver, magnesium, and strontium are reasonably employed in bone tissue engineering to endow implants with fine antibacterial properties. However, magnesium and strontium have not been investigated in detail. Silver has low biological activity and high cytotoxicity [45]. Copper ions can achieve a more satisfactory balance among antibacterial property, biological activity, and biocompatibility [46]. Compared with other antibacterial metals, the antibacterial concentration of Cu is lower than its toxic concentration to cells [47]. Milkovic et al. [48] investigated the proliferation of osteocytes on the bioglass surface containing varying concentrations of copper (0.1–12.5%) and found that 0.1% copper had no cytotoxicity. Liu et al. [49] verified the good cytocompatibility of copper-titanium alloy using an *in vitro* cell culture system. These results are thus consistent with our experimental findings.

### 4.6 Osteogenic differentiation

**Figure 9:**
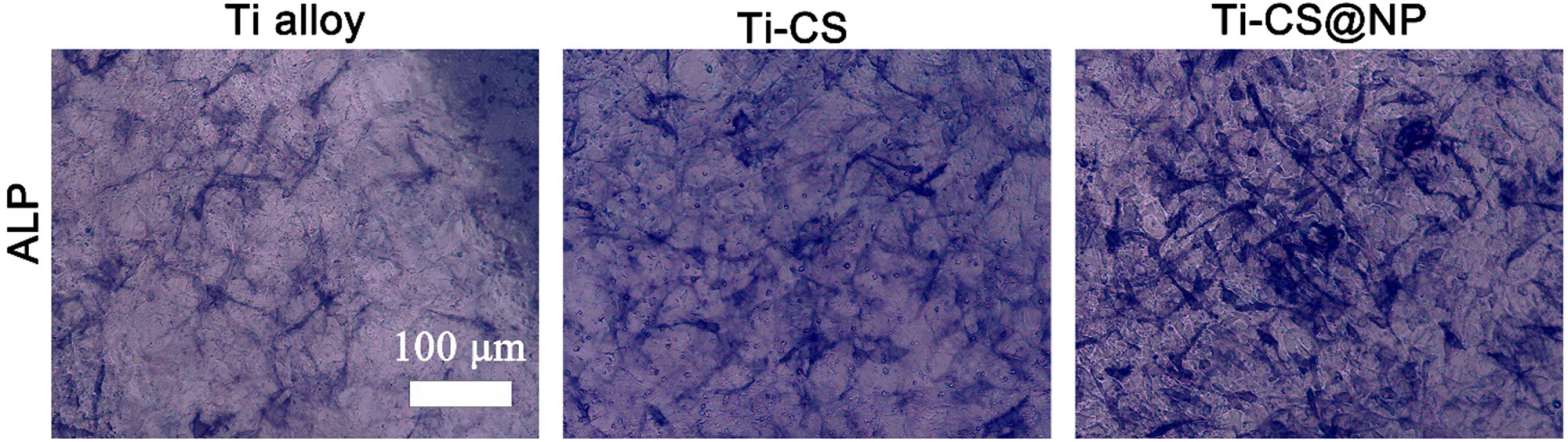
ALP staining of MC3T3-E1 cells cultured on the Ti alloy sheet sample surface (bare Ti alloy, Ti-CS, and Ti-CS@NP) for 7 days.

Alkaline phosphatase (ALP) is an external enzyme secreted by osteoblasts. Its main physiological function is to hydrolyze phosphate during osteogenesis, supply phosphate for HAP deposition, as well as hydrolyze pyrophosphate to remove its inhibitory effect on bone salt formation. ALP is an early indication of the osteogenic phenotype because it plays a crucial role in the early stages of bone matrix mineralization. Therefore, monitoring cellular ALP activity on different coating samples is an effective method to characterize the coatings’ osteogenic ability. As shown in Figure 9, the Ti-CS@NP group exhibited a greater ALP-positive staining area than the Ti alloy and Ti-CS groups after 7 days of cultivation. This proves that the composite coating of Cu^2+^/TA/HAP (CS@NP) has better osteogenesis property.

## 5 Conclusion

In this study, a composite coating comprising HAP, CS, TA, and Cu^2+^ (Cu^2+^/TA/HAP composite coatings) was prepared on the 3D-printed porous Ti alloy scaffolds surface via electrophoretic deposition. Using a standard plate count method and the live/dead bacteria staining method the CS@NP coating was found to have good antibacterial properties. SEM images of MC3T3.E1 cells on the Ti-CS@NP surface (Ti alloy sheet and 3D printed porous Ti alloy scaffolds) displayed more lamellipodia extensions and more cells adhered to the surface than those on the bare Ti alloy surface, clearly demonstrating the superior cytocompatibility of CS@NP coating. These findings were supported by the results of fluorescence staining with FITC phalloidin and DAPI. Live/dead staining of MC3T3.E1 cells on the Ti alloy sheet sample surface indicated that the composite coating had little cytotoxicity. Finally, the ALP assay revealed that the CS@NP coating had better osteogenesis potential. Thus, 3D-printed porous Ti alloy scaffolds coated with CS@NP might be a promising candidate for clinical use. Based on this study, we hope to find coatings with more accurate technical parameters and more suitable properties and provide better guidance and help for the application of implants in the biomedical field.
